# Plant performance on Mediterranean green roofs: interaction of species-specific hydraulic strategies and substrate water relations

**DOI:** 10.1093/aobpla/plv007

**Published:** 2015-01-20

**Authors:** Fabio Raimondo, Patrizia Trifilò, Maria A. Lo Gullo, Sergio Andri, Tadeja Savi, Andrea Nardini

**Affiliations:** 1 Dipartimento di Scienze Biologiche ed Ambientali, Università di Messina, via F. Stagno D'Alcontres 31, 98166 Messina, Italy; 2 Harpo seic verdepensile, Via Torino 34, 34123 Trieste, Italy; 3 Dipartimento di Scienze della Vita, Università di Trieste, via L. Giorgieri 10, 34127 Trieste, Italy

**Keywords:** Anisohydric, arbutus, drought stress, green roof, isohydric, Mediterranean region, sage

## Abstract

Recent studies have highlighted the ecological, economic and social benefits assured by green roof technology to urban areas. However, green roofs are very hostile environments for plant growth because of shallow substrate depths, high temperatures and irradiance and wind exposure. This study provides experimental evidence for the importance of accurate selection of plant species and substrates for implementing green roofs in hot and arid regions, like the Mediterranean area. Experiments were performed on two shrub species (*Arbutus unedo* L. and *Salvia officinalis* L.) grown in green roof experimental modules with two substrates slightly differing in their water retention properties, as derived from moisture release curves. Physiological measurements were performed on both well-watered and drought-stressed plants. Gas exchange, leaf and xylem water potential and also plant hydraulic conductance were measured at different time intervals following the last irrigation. The substrate type significantly affected water status. *Arbutus unedo* and *S. officinalis* showed different hydraulic responses to drought stress, with the former species being substantially isohydric and the latter one anisohydric. Both *A. unedo* and *S. officinalis* were found to be suitable species for green roofs in the Mediterranean area. However, our data suggest that appropriate choice of substrate is key to the success of green roof installations in arid environments, especially if anisohydric species are employed.

## Abstract (precis)

Mediterranean native shrubs can be very useful for green roofs in hot and arid regions. Our data show that both *Arbutus unedo* L. and *Salvia officinalis* L. could be successfully utilized, although the choice of species should be based on the drought-resistant strategy relative to the desired technical performance of the green roof. Moreover, substrate selection was found to have a crucial role in the success of green roof installations in the Mediterranean area.

## Introduction

Green roofs are engineered ecosystems designed to favour plant establishment on manufactured layers installed over rooftops, and typically comprise lightweight mineral substrate, drainage and moisture retention layers and a root-resistant waterproofing barrier (VanWoert *et al*. 2005; Berndtsson 2010). Modern green roofs were first developed in the 1960s in Germany and, over the last 15 years, this technology has received increasing attention in several countries of Northern and Central Europe, North America, Australia, Japan and China (Bowler *et al*. 2010; Dvorak and Volder 2010; Williams *et al*. 2010; Chen 2013). This renewed interest for green roofs is a consequence of recent experimental evidence highlighting the ecological, economic and social benefits provided by this technology to urban areas. In fact, green roofs have been reported to improve urban management of water runoff (e.g. Getter *et al*. 2007; Lundholm *et al*. 2010; MacIvor and Lundholm 2011; Nardini *et al*. 2012*a*), reduce the consumption of energy for thermal comfort of buildings (e.g. Theodosiou 2003; Sailor *et al*. 2008; Blanusa *et al*. 2013), mitigate the ‘urban heat island’ effect (Gill *et al*. 2007; Takebayashi and Moriyama 2007; Mackey *et al*. 2012), improve acoustic insulation (Van Renterghem and Botteldooren 2008, 2009), improve air (Rowe 2011) and water quality (Carter and Jackson 2007; Berndtsson 2010) and sequester CO_2_ (Getter *et al*. 2009; Li *et al*. 2010). Moreover, this technology could prove useful for recycling of waste materials (Solano *et al*. 2012; Mickovski *et al*. 2013) and might provide effective instruments to ameliorate the urban appeal, increase the number of recreational spaces and improve urban biodiversity (Brenneisen 2006; MacIvor and Lundholm 2011).

Green roofs are rather hostile environments for plant growth, because of shallow substrate, high temperatures and irradiance and wind exposure (Getter and Rowe 2008; Liu *et al*. 2012). In particular, structural features of buildings frequently require the use of reduced substrate depths, with predictable impacts on water availability to vegetation. This, in turn, limits the number of species that can thrive over green roofs, especially in hot and arid regions like Mediterranean countries (Fioretti *et al*. 2010; Nardini *et al*. 2012*b*), where drought, high irradiance and temperatures are common stress factors even for natural vegetation (Sánchez-Gómez *et al*. 2006; David *et al*. 2007; Nardini *et al*. 2014). Under these environmental conditions, the plants' growth over green roofs is particularly challenging and thus requires specific technological and ecophysiological strategies to improve plant survival (Dvorak and Volder 2013).

In particular, the selection of substrates with high water holding capacity and high amounts of water available to plants is apparently a key requirement to improve the performance of green roofs in arid climates. As an example, Farrell *et al*. (2012) reported a correlation between the survival rate of different succulent species under drought stress and the water holding capacity of different substrates. Similarly, Razzaghmanesh *et al*. (2014) reported significant effects of substrate type on growth and survival of different grass species native to the Australian flora. Moreover, improving water holding capacity of the substrate, amended with different materials, has been reported to be effective in increasing plant survival rates and ameliorating plant water status under drought conditions (Farrell *et al*. 2013; Papafotiou *et al*. 2013; Savi *et al*. 2014).

The selection of drought-resistant plant species is as important as substrate features in order to assure the success of green roofs in arid environments. Specific studies addressing the relative suitability of different plant species for green roof development have appeared in recent years (Dvorak and Volder 2010; MacIvor and Lundholm 2011; Cook-Patton and Bauerle 2012; Papafotiou *et al*. 2013; Van Mechelen *et al*. 2014), but the most commonly used species are still small succulents, mainly belonging to the genus *Sedum* (Snodgrass and Snodgrass 2006; Oberndorfer *et al*. 2007; Rowe *et al*. 2012). These are characterized by shallow roots, high drought tolerance and relatively fast propagation (Snodgrass and Snodgrass 2006; Getter and Rowe 2009; Farrell *et al*. 2012). In contrast, only few studies have explored the possibility to use alternative plant species over green roofs in arid regions, despite the high number (and drought adaptation) of species native to the Mediterranean region (Benvenuti and Bacci 2010; Papafotiou *et al*. 2013; Benvenuti 2014; Van Mechelen *et al*. 2014). In particular, the impressive heterogeneity in plant hydraulic strategies and water relations displayed by Mediterranean plants (Nardini *et al*. 2014; Vilagrosa *et al*. 2014) might represent an important resource for designing green roofs with specifically requested technical features. As an example, isohydric species that display tight stomatal control of transpiration might help to design green roofs with high resistance against drought, as well as with low irrigation requirements (Rowe *et al*. 2014). On the other hand, anisohydric species that maximize transpiration and photosynthesis while tolerating very negative water potential values might represent a more interesting choice in order to favour transpirational cooling of buildings (Schweitzer and Erell 2014) and/or improve the capacity of green roofs to intercept water during intense albeit sporadic rainfall events (Nardini *et al*. 2012*a*).

In the present study, we provide experimental evidence for the importance of substrate characteristics, with special reference to water retention properties, to assure sufficient water availability to plants over green roofs under drought stress conditions. Moreover, we provide insights into the importance of species-specific drought-resistance strategies and hydraulic properties for selecting Mediterranean native species best suited for specific technical functions and ecological requirements of green roofs. To this aim, experiments were performed using two Mediterranean shrub species: *Arbutus unedo* L. and *Salvia officinalis* L. *Salvia officinalis* (sage) is a perennial, evergreen, sub-shrub species widely naturalized even outside its original habitat. *Arbutus unedo* (arbutus) is an evergreen shrub or small tree widely distributed in the Mediterranean Basin (Pignatti 2002). Both species are well known for their drought tolerance, although a specific comparison of their hydraulic strategies has not been previously performed.

## Methods

Experiments were performed between May and July 2012 on 36 plants of *A. unedo* and 36 plants of *S. officinalis*. Plants were provided at the end of April 2012 by a local nursery and planted in 24 experimental green roof modules with dimensions 75 × 23 × 27 cm (i.e. 12 modules per species, 3 plants per module **[see Supporting Information]**). The modules were assembled with the SEIC^®^ extensive system (Harpo Spa, Trieste, Italy). The layering included a water retention geotextile (MediPro MP), a drainage and aeration element (MediDrain MD), a filtering layer (MediFilter MF 1) and 18 cm of one of the two different experimental substrates provided by SEIC. Species-specific modules were divided into two main categories on the basis of substrate type tested: substrate A and substrate B. In summary, six modules per species contained substrate A and six modules were filled with substrate B **[see Supporting Information]**.

Both substrates consisted of a mixture of mineral material (lapillus, pomix, zeolite) and organic material (peat) with grain size ranging from 0.05 to 20 mm. However, substrate A had a lower percentage of grain size ranging from 0.05 and 10 mm, higher electrical conductivity (20 versus 13 mS/m) and pH (8.9 versus 7.6) and lower percentage of organic matter (4.2 versus 6.2 %) than substrate B (Table 1, data kindly provided by SEIC).

**Table 1.** Percentage of different grain sizes, organic matter, porosity and values of electrical conductivity and pH of the two substrate types utilized (i.e. A and B). Data are kindly provided by SEIC.

	Substrate type A	Substrate type B
Grain size <0.05 (% m/m s.s.)	0	2
Grain size <0.55 (% m/m s.s.)	1	7
Grain size <0.25 (% m/m s.s.)	2	12
Grain size <0.50 (% m/m s.s.)	6	16
Grain size <1.00 (% m/m s.s.)	13	21
Grain size <2.00 (% m/m s.s.)	20	30
Grain size <5.00 (% m/m s.s.)	50	53
Grain size <10.00 (% m/m s.s.)	93	100
Grain size <16.00 (% m/m s.s.)	99	100
Grain size <20 (% m/m s.s.)	100	100
Organic matter (% s.s.)	4.3	6.2
Porosity (% v/v)	65.9	65.7
Electrical conductivity (mS/m s.s.)	20	13
pH	8.9	7.6

The water retention properties of the two substrates were preliminarily measured using a dewpoint potentiameter (WP4, Decagon Devices, Pullman, WA, USA). In particular, the relationships between water content (WC) and water potential (pressure–volume curve) of the two substrates were measured to estimate the amount of water available to plants (Whalley *et al*. 2013). Samples of the two substrates were watered to saturation. After complete drainage of excess water, small samples (a few grams each) were collected and placed in dedicated WP4 sample-holders. Water potential of substrate (Ψ_s_) was measured in the continuous mode and after each reading, samples were weighed with an electronic balance (Basic BA110S, Sar-torius AG, Göttingen, Germany) to obtain their fresh weight (FW), and then oven-dried at 70 °C for 24 h. Samples were weighed again to get their dry weight (DW). Water content of samples was calculated as (FW − DW)/DW. Measurements were performed on fully hydrated samples as well as on samples air-dehydrated for increasing time intervals.

Green roof modules were randomly located over the flat rooftop of the Department of Biological and Environmental Sciences, University of Messina. On the basis of irrigation regime, experimental modules were further divided in four experimental groups per species **[see Supporting Information]**: three modules per substrate type category were regularly watered to field capacity (well-watered plants: WA and WB), while the other three modules per substrate-type category received irrigation up to 75 % field capacity (stressed plants: SA and SB). Irrigation was supplied at 48 h intervals for 10 weeks. At the end of the treatment, all plants were irrigated to field capacity and physiological measurements were performed again 24 and 48 h after irrigation.

During the study period, mean air temperatures and relative humidity in the area were 19 ± 1 °C and 74 ± 7 % in May, 24 ± 2 °C and 75 ± 5 % in June and 28 ± 1 °C and 74 ± 5 % in July, respectively. The total rainfall was 13 mm only. Climatic data were obtained from the weather station of Torre Faro, Messina, Italy.

At the beginning and at the end of the experiment (i.e. beginning of May and end of July, respectively), two plants within each module of *S. officinalis* and two plants within each module of *A. unedo* per each experimental group (i.e. WA, SA, WB and SB) were selected and the following parameters were measured: plant height (H), trunk diameter at the root-stem transition zone (Ø) and total number of leaves per plant (*N* leaves/plant). During the study period, substrate water status (Ψ_s_) of both W and S-modules was estimated by measuring the pre-dawn water potential (Ψ_pd_) of six leaves wrapped in cling-film the day before measurements (two leaves per species and per module) and sampled at 0500 hours (solar time). Measurements were performed with a pressure chamber (3005 Plant Water Status Console, Soilmoisture Equipment Corp., Goleta, CA, USA), assuming that under nocturnal low transpiration conditions leaf water potential equilibrated with Ψ_s_, so that Ψ_pd_ ∼ Ψ_s_ (Richter 1997; Nardini *et al*. 2003). The indirect estimation of Ψ_s_ was preferred to direct sampling of the substrate, in order to avoid the risk of damage to the root system. Measurements of Ψ_pd_ were performed on the same days selected for gas exchange and midday leaf water potential measurements (see below).

### Measurements of leaf gas exchange and water status

At the end of the 10-week treatment period, both 24 and 48 h after irrigation, maximum leaf stomatal conductance to water vapour (*g*_L_) and transpiration rate (*E*_L_) were measured between 1200 and 1400 hours on leaves of at least one plant per module per experimental group and species using a steady-state porometer (LI-1600, LICor Inc., Lincoln, NE, USA). At the same time, midday diurnal leaf water potential (Ψ_midday_) was estimated using a portable pressure chamber (3005 Plant Water Status Console, Soilmoisture Equipment Corp.).

In order to quantify eventual acclimation of water relation parameters in terms of leaf water potential at the turgor loss point (Ψ_tlp_), osmotic potential at full turgor (*π*_0_) and bulk modulus of elasticity (*ɛ*_max_), leaf water potential isotherms of leaves of at least one plant per module per experimental group were determined from pressure–volume (P–V) curves (Tyree and Hammel 1972). Measurements were performed before starting the treatment and repeated at the end of the 10-week period, respectively.

### Estimating plant hydraulic conductance (*K*_plant_)

Whole-plant hydraulic conductance (*K*_plant_) was estimated *in planta* using the Evaporative Flux Method on at least one plant per module per species and per experimental group (Nardini *et al*. 2003). *K*_plant_ was calculated as: *E*_L_/(Ψ_midday_ − Ψ_s_), where *E*_L_, Ψ_midday_ and Ψ_s_ were measured as described above. All hydraulic conductance values were corrected to a temperature of 20 °C, to take into account changes in water viscosity.

### Statistical analysis

Data were analysed with the SigmaStat 2.0 (SPSS, Inc., Chicago, IL, USA) statistics package. To test the differences among substrate type and the effects of both irrigation regimes and time after last irrigation on Ψ_s_, *g*_L_ and *K*_plant_, a three-way ANOVA was performed (soil, irrigation and time as factors) with Type III sums of squares. The same test was used to check the significance of the differences among substrate type and the effects of irrigation regime and time (i.e. May and July) on H, Ø and N leaves/plant. To test the differences among substrate type and effects of irrigation regime on Ψ_tlp_, *π*_o_ and *ɛ*_max_ a two-way ANOVA test was performed. Data have been analysed by nesting the plant observations within each module (*n* = 3).

When the difference was significant, a post hoc Tukey's test was carried out. Relationships between the studied characteristics and independent variables were assessed by Pearson's correlations.

## Results

Both irrigation regime and measurement time influenced plant size, as estimated in terms of final plant height and number of leaves per plant in *S. officinalis* but not in *A. unedo* plants (Tables 2 and 4). In fact, in well-watered sage samples (WA and WB), plant height was ∼26 cm in May, and increased to ∼40 cm by the end of the experimental treatment. In contrast, the size of stressed samples increased by only less than ∼30 cm. A different trend was recorded in *A. unedo* plants, where an increase of ∼25 % in terms of plant height was recorded after 10 weeks in all experimental groups, with no effect of irrigation regime. The increase in the number of leaves per plant during the study period was larger in *S. officinalis* than in *A. unedo*, both in well-watered (+100 % versus about +60 %, respectively) and stressed samples (see below). Moreover, in *S. officinalis* as well as in *A. unedo* the number of leaves per plant was influenced by irrigation regime and time.

**Table 2.** Means ± SD (*n* = 3) of plant height (H), trunk diameter (Ø) and number of leaves per plant (*N* leaves/plant) as recorded in May and in July (i.e. at the beginning and at the end of treatment irrigation regimes) in plants of *S. officinalis* and *A. unedo* growing in two types of substrate (A and B) and irrigation regimes (W: plants irrigated to field capacity; S: plants irrigated to 75 % field capacity) (for details, see text). Different letters indicate, for each measured parameter, statistically different mean values for Tukey pairwise comparison, after performing a three-way ANOVA test.

	WA		WB		SA		SB	
	May	July	May	July	May	July	May	July
*S. officinalis*								
H (cm)	25.8 ± 1.4c	39.0 ± 2.0a	26.6 ± 1.3c	40.7 ± 3.7a	26.7 ± 1.2c	29.9 ± 2.0b	26.0 ± 2.5c	30.4 ± 2.2b
Ø (cm)	0.6 ± 0.005b	0.8 ± 0a	0.6 ± 0.007b	0.8 ± 0.007a	0.6 ± 0.01b	0.8 ± 0.003a	0.6 ± 0.006b	0.8 ± 0a
*N* leaves/plant	94 ± 4.2c	195 ± 12a	94 ± 3.6c	197 ± 8a	100 ± 7c	155 ± 6b	94 ± 3c	142 ± 7b
*A. unedo*								
H (cm)	43 ± 1.2b	49.3 ± 0.6a	42.5 ± 1.6b	49.7 ± 1.3a	41.7 ± 1.6b	48.8 ± 1.0a	43.3 ± 0.6b	49.8 ± 1.3a
Ø (cm)	0.5 ± 0.005b	0.7 ± 0.005a	0.5 ± 0.005b	0.7 ± 0.002a	0.5 ± 0.002b	0.7 ± 0.03a	0.5 ± 0.01b	0.7 ± 0.008b
*N* leaves/plant	102 ± 1c	162 ± 3a	102 ± 1c	158 ± 4a	104 ± 1c	128 ± 2b	104 ± 1c	128 ± 1b

**Figure 1.**
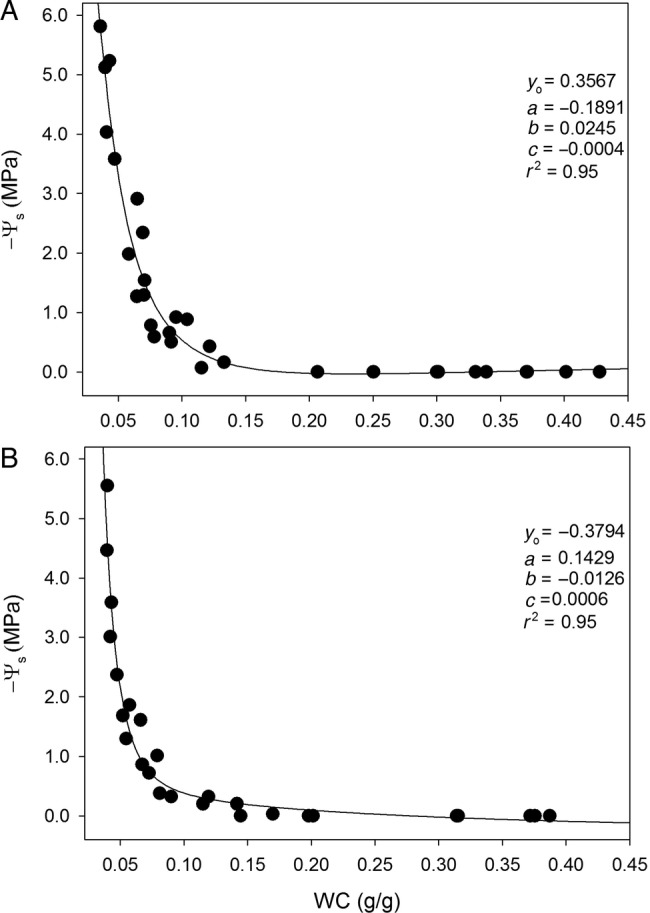
Relationships between water potential (−Ψ_s_) and water content (WC) as measured for the substrate A (A) and B (B). Regression curves are expressed by the following function: *f* = *y*_0_ + (*a*/*x*) + (*b*/*x*^2^) + (*c*/*x*^3^). Coefficient values and correlation coefficients (*r*^2^) are reported.

Figure 1 reports the relationship between soil water potential and WC as measured for substrates A and B. Water content at saturation (SWC) was ∼0.43 g g^−1^ for substrate A and 0.39 g g^−1^ for substrate B. At Ψ_s_ = −1.5 MPa (i.e. the reference value of permanent wilting point, WWC), WC was ∼0.07 g g^−1^ for both substrate types. Hence, the amount of water available to plants (AWC) calculated as SWC—WWC turned out to be ∼12 % higher in substrate A (0.36 g g^−1^) than in substrate B (0.32 g g^−1^).

**Figure 2.**
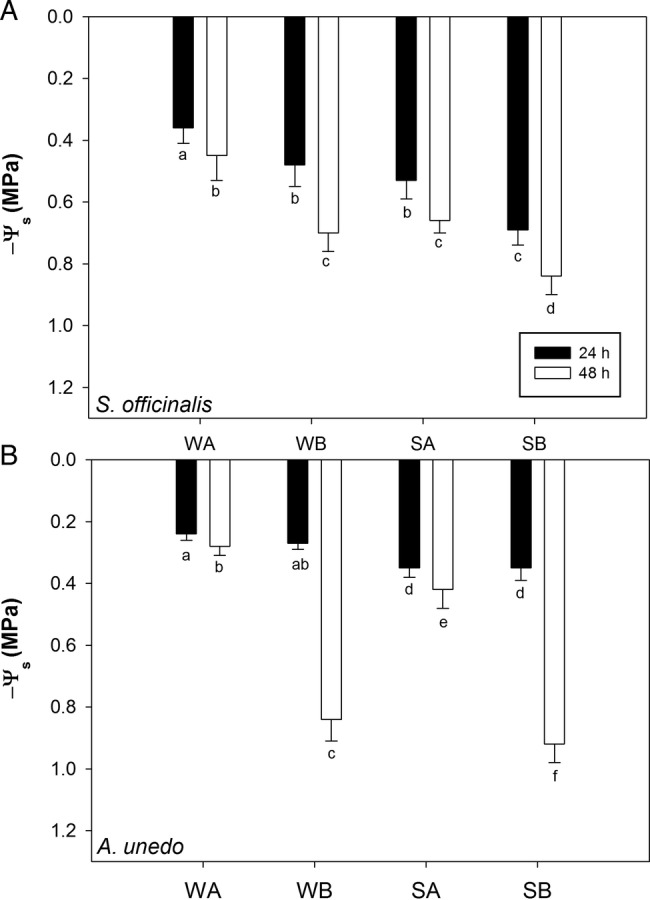
Substrate water potential (Ψ_s_) as recorded 24 and 48 h after irrigation of experimental modules with *S. officinalis* (A) and *A. unedo* (B) plants subjected to two irrigation regimes (W: plants irrigated to field capacity; S: plants irrigated to 75 % field capacity). Two substrates were tested (A and B, for details, see text). Means are given ±SD (*n* = 3). Different letters indicate statistically different mean values for Tukey pairwise comparison.

**Figure 3.**
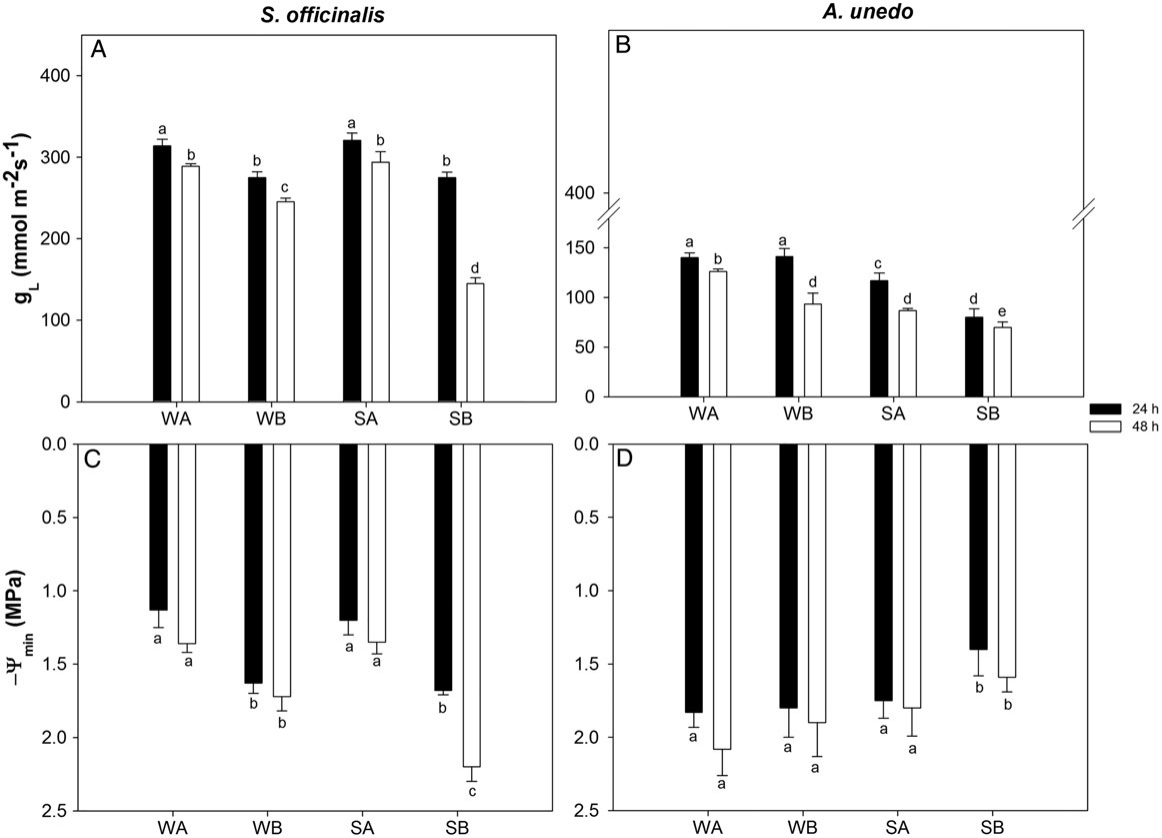
Leaf conductance to water vapour (*g*_L_, A and B) and leaf water potential (Ψ_midday_, C and D) as recorded in plants of *S. officinalis* and *A. unedo* growing in the two types of substrate (A and B) and under different irrigation regimes (W: plants irrigated to field capacity; S: plants irrigated to 75 % field capacity, for details, see text). Means are given ±SD (*n* = 3). Different letters indicate statistically significant differences for Tukey pairwise comparison.

In accordance with the above-reported differences in terms of SWC and AWC, larger drops of Ψ_s_ were recorded within 48 h in modules containing substrate B than modules filled with substrate A, irrespective of the species (Fig. 2). In detail, Ψ_s_ values, as recorded 48 h after irrigation, were about −0.5 and −0.7 MPa in WA and SA sage plants, respectively, while values of about −0.7 and −0.9 MPa were recorded in WB and SB samples. Likewise, in WA and SA arbutus plants, 48 h after last irrigation, Ψ_s_ values of about −0.3 and −0.5 MPa were recorded in WA and SA samples and values of about −0.9 and −1.0 MPa were found in WB and SB ones. Midday *g*_L_ values recorded in *S. officinalis* growing in modules containing substrate A were higher than values recorded in samples growing in modules containing substrate B, as recorded 24 h after last irrigation (i.e. ∼300 mmol m^−2^ s^−1^ versus ∼270 mmol m^−2^ s^−1^). Moreover, while in WA, WB and SA samples stomatal conductance decreased no more than ∼10 % within 48 h after last irrigation, in SB samples a decrease of ∼50 % of *g*_L_ values was recorded 48 h after last irrigation (Fig. 3A). A different trend was recorded in arbutus plants (Fig. 3B) where in samples growing in substrate A, *g*_L_ decreased by ∼10 % in well-watered samples and by ∼20 % in stressed samples. In WB arbutus plants *g*_L_ decreased by ∼40 % 48 h after last irrigation with respect to values recorded 24 h before. Moreover, SB samples showed values of *g*_L_ of ∼80 mmol m^−2^ s^−1^ 24 h after the last irrigation, and further decreasing to ∼70 mmol m^−2^ s^−1^ 48 h after last irrigation. A contrasting behaviour was observed in *S. officinalis* and *A. unedo* also in terms of changes in leaf water potential. In WA and SA sage plants, Ψ_midday_ showed similar values (i.e. about −1.25 MPa) and remained quite constant over 48 h after last irrigation (Fig. 3C). In contrast, Ψ_midday_ measured in WB and SB samples was about −1.7 MPa in both experimental groups 24 h after last irrigation and, 48 h after last irrigation, midday leaf water potential values remained quite constant in WB plants while decreased to about −2.3 MPa in SB samples. In arbutus plants, Ψ_midday_ was maintained constantly around −1.8 MPa in all treatments except in SB samples where values of about −1.5 MPa were recorded (Fig. 3D).

All recorded Ψ_midday_ values were within the positive turgor region (Table 2). However, midday leaf water potential of sage plants growing in substrate B was close to the critical turgor loss point. In fact, Ψ_tlp_ values of W and S sage samples were about −1.8 and −2.3 MPa, respectively. However, in WA and SA samples, Ψ_midday_ values no lower than about −1.3 MPa were recorded while in WB and SB samples Ψ_midday_ values were low as about −1.72 MPa and about −2.2 MPa, respectively (Fig. 3C). In arbutus plants, Ψ_tlp_ was −2.4 ± 0.1 and −2.6 ± 0.01 MPa in WA and WB treatments, respectively, and about −3 MPa in S samples, whereas Ψ_midday_ remained above −2.0 MPa (Fig. 3D). Changes in Ψ_tlp_ in watered and stressed plants as recorded in both species under study were apparently driven by changes in different parameters. Irrigation regimes, in fact, significantly affected only *π*_0_ values in sage plants, while more apparent changes in *ɛ*_max_ values were recorded in arbutus plants (Table 3).

**Table 3.** Leaf water potential at turgor loss point (Ψ_tlp_), osmotic potential at full turgor (*π*_0_) and bulk modulus of elasticity (*ɛ*_max_) as recorded in plants of *S. officinalis* and *A. unedo* growing in two types of substrate (A and B) and irrigation regimes (W: plants irrigated to field capacity; S: plants irrigated to 75 % field capacity) (for details, see text). Means are given ±SD (*n* = 3). Different letters indicate, for each measured parameter, statistically different mean values for Tukey pairwise comparison, after performing a three-way ANOVA test.

	*S. officinalis*			*A. unedo*		
	−Ψ_tlp_ (MPa)	−*π*_o_ (MPa)	*ɛ*_max_ (MPa)	−Ψ_tlp_ (MPa)	−*π*_o_ (MPa)	*ɛ*_max_ (MPa)
WA	1.61 ± 0.01a	1.36 ± 0.14a	11.35 ± 1.4	2.41 ± 0.1a	1.96 ± 0.2	22.95 ± 1.8b
WB	1.84 ± 0.13a	1.49 ± 0.09a	13.20 ± 1.1	2.61 ± 0.01a	2.20 ± 0.2	25.30 ± 3.0b
SA	2.40 ± 0.13b	1.73 ± 0.08b	13.03 ± 1.1	2.92 ± 0.03b	2.17 ± 0.2	31.85 ± 1.1a
SB	2.29 ± 0.16b	1.83 ± 0.04b	11.73 ± 1.2	3.03 ± 0b	2.11 ± 0.07	34.75 ± 2.0a

**Figure 4.**
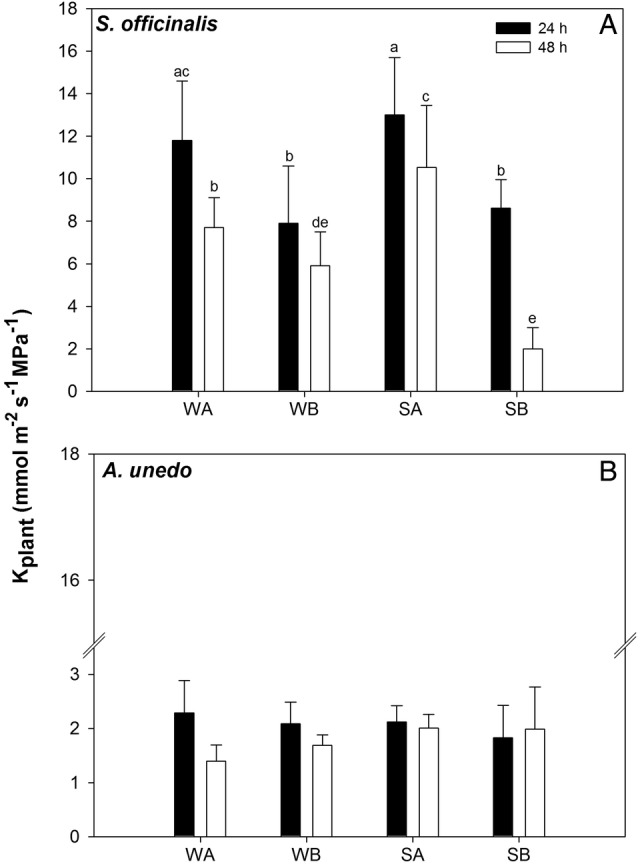
Plant hydraulic conductance (*K*_plant_) as recorded in plants of *S. officinalis* (A) and *A. unedo* (B) growing in two types of substrate (A and B) and under different irrigation regimes (W: plants irrigated to field capacity; S: plants irrigated to 75 % field capacity, for details, see text). Means are given ±SD (*n* = 3). Different letters indicate statistically significant differences for Tukey pairwise comparison.

*K*_plant_ values changed in response to both type of substrate and time after last irrigation in *S. officinalis* samples (Fig. 4A, Table 4). In WA and SA sage samples and in WB and SB plants, *K*_plant_ decreased over 48 h after the last irrigation. However, 24 h after last irrigation, plants growing in modules containing substrate B showed values of *K*_plant_ lower than samples growing in modules containing substrate A (i.e. ∼8 versus ∼12 mmol m^−2^ s^−1^ MPa^−1^, respectively). In arbutus, *K*_plant_ was maintained at a constant value of ∼2 mmol m^−2^ s^−1^ MPa^−1^ in all treatments over 48 h after the last irrigation (Fig. 4B).

**Table 4.** Results of: (A) a three-way ANOVA of different measured parameters by soil type, *S* (i.e. A and B), irrigation regime, *I* (i.e. samples regularly watered to field capacity and samples watered to 75 % field capacity) and time, *T* (i.e. time after last irrigation for soil water potential Ψ_s_, maximum diurnal leaf conductance to water vapour *g*_L_, minimum diurnal leaf water potential Ψ_min_ and plant hydraulic conductance *K*_plant_, and time of year for plant height *H*, stem diameter Ø and number of leaves per plant *N* leaves/plant) treatments; (B) a two-way ANOVA of parameters determined from P–V curves by soil type, S (i.e. A and B) and irrigation treatment, I (i.e. time of the year) recorded in *S. officinalis* and in *A. unedo*. For details, see the text. Numbers represent *F* values, *P < 0.05, **P < 0.01; ***P < 0.001.

	*S*	*I*	*T*	*S* × *I*	*S* × *T*	*T* × *I*	*S* × *T* × *I*
(A)							
*S. officinalis*							
Ψ_s_	52.6***	55.3***	35.2***	0.04	2.05	0.074	1.19
*g*_L_	477.5***	47.87***	274.86***	79.26***	71.11***	64.25***	57.72***
Ψ_min_	213.9***	15.88***	42.55***	9.44**	2.43	5.36*	11.3**
*K*_plant_	31.03***	0.061	20.61***	4.65*	0.366	0.791	3.532
H	0.37	28.79***	91.59***	0.417	0.417	29.19***	0.0003
Ø	0.714	1.4	6555.46***	0.714	1.4	0.714	0.257
*N* leaves/plant	2.06	61.4***	701.43***	2.915	0.25	76.66***	0.533
*A. unedo*							
Ψ_s_	219.1***	31.3***	287.9***	1.597	193.2***	0.13	0.033
*g*_L_	58.4***	170.67***	84.15***	3.65	1.44	3.38	23.32***
Ψ_min_	13.98**	6.75*	3.07	1.19	0.101	0.133	0.195
*K*_plant_	0.07	0.378	2.602	0.289	0.97	3.005	0.088
H	1.37	0.314	180.3***	1.873	0.033	0.00109	0.55
Ø	0.128	3.872	1889.6***	0.512	2.048	0.032	3.2
*N* leaves/plant	1.305	275.09***	2000.92***	0.603	1.305	366.51***	1.3
	***S***	***I***	***S × I***				
(B)							
*S. officinalis*							
Ψ_tlp_	0.149	29.8***	2.11				
*π*_ο_	4.19	40.69***	0.071				
*ɛ*_max_	0.182	0.0282	5.97*				
*A. unedo*							
Ψ_tlp_	5.98	85.09***	0.591				
*π*_ο_	1.17	0.293	2.635				
*ɛ*_max_	3.87	55.93***	0.125

**Figure 5.**
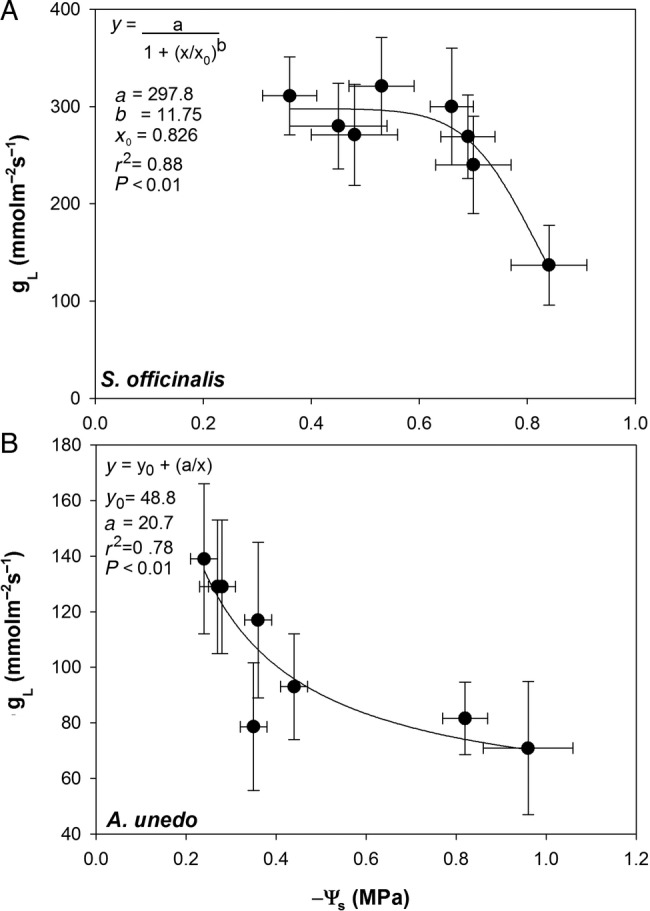
Relationship between maximum leaf stomatal conductance to water vapour (*g*_L_) values and substrate water content (Ψ_s_) values recorded in plants of *S. officinalis* (A) and *A. unedo* (B) growing in two types of substrate and under different irrigation regimes. Regression equation, coefficient values, *P*-values and correlation coefficients (*r*^2^) are also reported.

**Figure 6.**
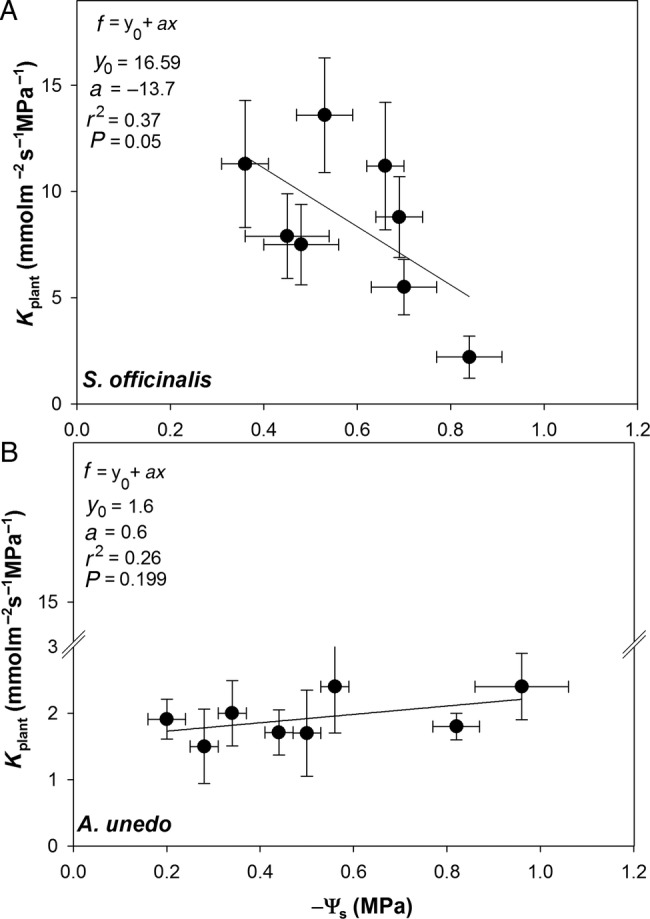
Relationship between plant hydraulic conductance (*K*_plant_) values and corresponding substrate water status (Ψ_s_) recorded in plants of *S. officinalis* (A) and *A. unedo* (B) growing in the two types of substrate and subjected to different irrigation regimes. Regression equation, coefficient values, *P*-values and correlation coefficients (*r*^2^) are also reported.

When *g*_L_ values were plotted versus the corresponding Ψ_s_, different relationships were observed in sage and arbutus plants (Fig. 5). In sage plants, *g*_L_ values remained quite constant until Ψ_s_ was above −0.6 MPa. In contrast, in arbutus plants, *g*_L_ was related to Ψ_s_ according to an inverse first-order polynomial equation. Likewise, different values of *K*_plant_ as a function of Ψ_s_ were recorded in sage plants, while a constant water transport efficiency from root to leaves was recorded in arbutus plants, despite the treatments (Fig. 6).

## Discussion

Our data suggest that the use of species selected from the native flora of the Mediterranean region might be a valuable strategy for implementation of green roof systems in hot and arid areas. On the other hand, our findings reveal that even subtle differences in terms of substrate properties, with special reference to water relation parameters, can have very important consequences for the performance and persistence of vegetation over green roofs.

Substrate A was more suitable than substrate B for installation of efficient and fully functional green roofs in arid-prone areas. This was mainly due to the higher water retention capability related to the particle size, and especially to the higher amounts of water potentially available to plants (Fig. 1). This feature resulted in the maintenance of higher soil water potential values over 48 h after the last irrigation in plants growing in modules containing substrate A than in samples growing in modules filled with substrate B, as observed in both species, despite their different water relations strategies (Figs 2 and 3).

Arbutus and sage plants apparently adopted contrasting strategies to cope with drought stress. On the basis of relationships between *g*_L_ and leaf water potential, it can be suggested that *A. unedo* adopted a rather typical isohydric behaviour, while *S. officinalis* displayed a significant level of anisohydry, although a recent study has highlighted the fact that there might be a continuum of water relations strategies along these two ideal extremes (Klein 2014). Values of *g*_L_ were lower in arbutus than in sage, even in well-watered samples (∼130 versus 300 mmol m^−2^ s^−1^, respectively, Fig. 3A and B), and a further reduction of stomatal conductance was observed in arbutus plants under water stress (∼70 mmol m^−2^ s^−1^). Progressive stomatal closure apparently allowed arbutus plants to limit water loss and maintain relatively stable leaf water potential values both under well-watered and drought stress conditions, especially in samples growing in modules filled with substrate type A (Figs 3D and 5B). In contrast, *S. officinalis* plants maintained values of *g*_L_ as high as ∼300 mmol m^−2^ s^−1^ as long as soil water potential remained above a critical value of about −0.6 MPa (Figs 3C and 5A). Below this threshold, gas exchange rates were reduced by ∼50 % (from 300 to 150 mmol m^−2^ s^−1^, as recorded in SB samples 48 h after last irrigation Fig. 3A). This, in turn, induced statistically significant differences in leaf water potential values as a function of the time after the last irrigation, regime of irrigation and the type of substrate (Fig. 3C, Table 4). The different water use strategies adopted by arbutus and sage plants to face drought stress were also confirmed by the analysis of leaf water potential isotherms. In fact, water-stressed plants of *S. officinalis* lowered the leaf water potential at the turgor loss point by osmotic adjustment. In the case of arbutus, water stress induced a significant increase of the bulk modulus of elasticity (*ɛ*_max_, Tables 3 and 4).

Isohydric and anisohydric behaviour of different species/genotypes could arise from different stomatal sensitivity to xylem-born ABA (Tardieu and Simonneau 1998; Beis and Patakas 2010; Gallè *et al*. 2013) and/or to different levels of xylem hydraulic safety/efficiency (Schultz 2003; Tombesi *et al*. 2014). Different levels of stomatal control of transpiration under drought stress are known to affect photosynthetic productivity and plant growth (Medrano *et al*. 2002; Xu and Zhou 2008). In the present study, the anisohydric behaviour recorded in sage plants was coupled to a strong reduction of the number of leaves per plant as recorded in July in stressed versus watered samples (i.e. ∼100 % versus ∼40 %). Isohydric and anisohydric behaviours of the two study species were further supported by estimates of plant hydraulic conductance (Fig. 5). In fact, arbutus plants (isohydric) showed three times lower *K*_plant_ than sage plants (anisohydric, Fig. 4), and this parameter remained quite constant up to 48 h after the last irrigation in samples growing in modules filled with substrate B, despite wide variations in terms of soil water availability (Figs 2B, 4B and 5B). In contrast, *K*_plant_ of *S. officinalis* strongly changed as a function of Ψ_s_ (Figs 4A and 5A). In other words, the isohydric behaviour of arbutus allowed to maintain stable *K*_plant_ values, while anisohydry in sage implied a drop of *K*_plant_ as drought progressed.

## Conclusions

Data recorded in the present study suggest that arbutus plants could overcome intense drought conditions and, then, might be more suitable for Mediterranean green roofs than to sage plants. In fact, the higher water use of the latter species might imply the need of additional irrigation to prevent foliage damage and/or desiccation under prolonged drought. In the literature, *A. unedo* is frequently reported to be able to survive even severe drought stress (i.e. Castell and Terradas 1995; Gratani and Ghia 2002; Munné-Bosch and Peñuelas 2004), as it apparently maintains a positive carbon balance until pre-dawn leaf water potential values of −4 MPa (Filella and Peñuelas 2003). In contrast, sage plants are known to show leaf senescence symptoms when exposed to severe drought conditions (i.e. Ψ_pd_ < −1 MPa, Munné-Bosch *et al*. 2001; Abreu and Munné-Bosch 2008; Savi *et al*. 2013). Hence, while arbutus might represent a suitable species for green roofs with very low input of additional irrigation, sage might be more recommendable in order to maximize the transpirational cooling of buildings and/or to favour fast water depletion from substrates, thus improving the effectiveness of green roofs to mitigate water runoff during occasional storms, although the use of this species would probably be possible only when regular albeit low irrigation inputs are guaranteed (Savi *et al*. 2013). Additional studies focussed on testing the physiological performance and water requirements of a large number of Mediterranean species over green roofs are required to conclude about possible relationships between plant hydraulic strategies and green roof performance under drought.

## Sources of Funding

This work was supported by University of Messina (Atheneum Research Project). Materials of set up of green roof experimental modules were kindly provided by Harpo Spa (Trieste, Italy).

## Contributions by the Authors

P.T., M.A.L.G., S.A. and A.N. designed the experiment and planned the measurements. F.R., P.T., S.A. and T.S. carried out experiments. F.R. and P.T. analysed the data. P.T. wrote the manuscript. M.A.L.G., A.N. and T.S. revised and finalized the manuscript. All authors read and approved the final manuscript.

## Conflicts of Interest Statement

None declared.

## Supporting Information

The following additional information is available in the online version of this article –

**Figure S1.** Schematic representation of the experimental design. Twenty-four modules (75 × 23 × 27 cm) were divided in two groups of 12 modules in which 36 plants of *A. unedo* and 36 plants of *S. officinalis* were planted, respectively (i.e. 3 plants per module). Two types of substrate (A and B) and two irrigation regimes (well watered, W and stressed, S) were tested. More in detail, 12 modules per species were divided in two categories on the basis of substrate type tested: 6 modules per species contained substrate A and the other 6 modules contained substrate B. These modules were further divided in four experimental groups on the basis of irrigation regime: 3 modules per substrate type category were regularly watered to field capacity (i.e. WA and WB modules), and 3 modules per substrate type category received irrigation up to 75 % field capacity (i.e. SA and SB modules).

Additional Information
