# Supplementary material for: Plant performance on Mediterranean green roofs: interaction of species-specific hydraulic strategies and substrate water relations
Source: AoB Plants. 2015 Jan 20;7:plv007. doi: 10.1093/aobpla/plv007 (PMC4344481; doi:10.1093/aobpla/plv007)
Supplement: Additional Information [file supp_7_plv007_index.html]

Plant performance on Mediterranean green roofs: interaction of species-specific hydraulic strategies and substrate water relations — Additional Information 

# Plant performance on Mediterranean green roofs: interaction of species-specific hydraulic strategies and substrate water relations

## Additional Information

Additional Information

**Files in this Data Supplement:**

- Supplementary Figure 1 - Doc file
