# Supplementary material for: Plant performance on Mediterranean green roofs: interaction of species-specific hydraulic strategies and substrate water relations
Source: AoB Plants. 2015 Jan 20;7:plv007. doi: 10.1093/aobpla/plv007 (PMC4344481; doi:10.1093/aobpla/plv007)
Supplement: Additional Information [file supp_plv007_plv007supp.doc]

Figure S1: Schematic representation of the experimental design. 24 modules (75 x 23 x 27 cm) were divided in two groups of 12 modules in which 36 plants of A. undeo and 36 plants of S. officinalis were planted, respectively (i.e 3 plants per module). Two type of soils (A and B) and two irrigation regimes (well watered, W and stressed, S) were tested. 12 modules per species were divided in two categories on the basis of substrate type tested: 6 modules per species containing substrate A and the other 6 modules containing substrate B. And, then, they were further divided in four experimental groups on the basis of irrigation regime: 3 modules per substrate type category and regularly watered to field capacity (i.e. WA and WB modules), and 3 modules per substrate type category and receiving irrigation up to 75% field capacity (i.e. SA and SB modules).

**24 modules**

**12 species-specific modules**

(36 plants of *A. unedo, i.e.* 3 plants per module)

**6 substrate A**

**modules**

**6 substrate A**

**modules**

**6 substrate B**

**modules**

**6 substrate B**

**modules**

**3 SA**

**modules**

(9 plants)

**3 WA**

**modules**

(9 plants)

**3 WA**

**modules**

(9 plants)

**3 WA**

**modules**

(9 plants)

**3 WA**

**modules**

(9 plants)

**3 SA**

**modules**

(9 plants)

**3 SA**

**modules**

(9 plants)

**3 SA**

**modules**

(9 plants)

**12 species-specific modules**

(36 plants of *S. officinalis, i.e.* 3 plants per module)
